# Micromechanics of Sea Urchin Spines

**DOI:** 10.1371/journal.pone.0044140

**Published:** 2012-09-11

**Authors:** Naomi Tsafnat, John D. Fitz Gerald, Hai N. Le, Zbigniew H. Stachurski

**Affiliations:** 1 School of Mechanical and Manufacturing Engineering, University of New South Wales, Sydney, New South Wales, Australia; 2 Research School of Earth Sciences, Australian National University, Canberra, Australian Capital Territory, Australia; 3 Research School of Engineering, Australian National University, Canberra, Australian Capital Territory, Australia; University of Zurich, Switzerland

## Abstract

The endoskeletal structure of the Sea Urchin, *Centrostephanus rodgersii*, has numerous long spines whose known functions include locomotion, sensing, and protection against predators. These spines have a remarkable internal microstructure and are made of single-crystal calcite. A finite-element model of the spine’s unique porous structure, based on micro-computed tomography (microCT) and incorporating anisotropic material properties, was developed to study its response to mechanical loading. Simulations show that high stress concentrations occur at certain points in the spine’s architecture; brittle cracking would likely initiate in these regions. These analyses demonstrate that the organization of single-crystal calcite in the unique, intricate morphology of the sea urchin spine results in a strong, stiff and lightweight structure that enhances its strength despite the brittleness of its constituent material.

## Introduction

The endoskeletal structure of the purple-spined Sea Urchin *Centrostephanus rodgersii* from the New South Wales coast of Australia has on its outside long and numerous spines whose functions include locomotion, sensing, and protection from physical trauma and predators [1], [2]. The spines protect the spherical test, often by “sacrificing” themselves to absorb energy as they break [1]. In the case of attack by a predator, or impact by an object in surf conditions, the spines can protect the test in two ways. If a predator impacts axially, the spine pierces the object and snaps off, requiring high strength in compression, and brittle fracture in tension or torsion. If an object impacts the spine along its length, it absorbs the energy by brittle fracture in bending. In both cases the energy is absorbed and the load is spread away from the test. Other functions of the spines include locomotion and sensing, which would place significantly less stress on them than impact. For these functions, the spines would need to be axially stiff with enough elasticity to withstand loads in a high energy ocean surge environment.

Sea Urchin spines are made of a single crystal of calcite with the crystallographic c-axis along the spine's length [3]. A monolithic structure comprised of a single crystal of calcite would be very brittle, however Urchin spines are relatively flexible and this has been attributed to a small amount of glycoprotein embedded in the mineral phase that enhances their fracture resistance and increases their elastic limit [3]–[5]. An intimate mixture of organic and mineral matter in the form of an “oriented array of nanocrystals” has also been used to explain other remarkable properties of urchin-spine biomaterial [6].

**Figure 1:**
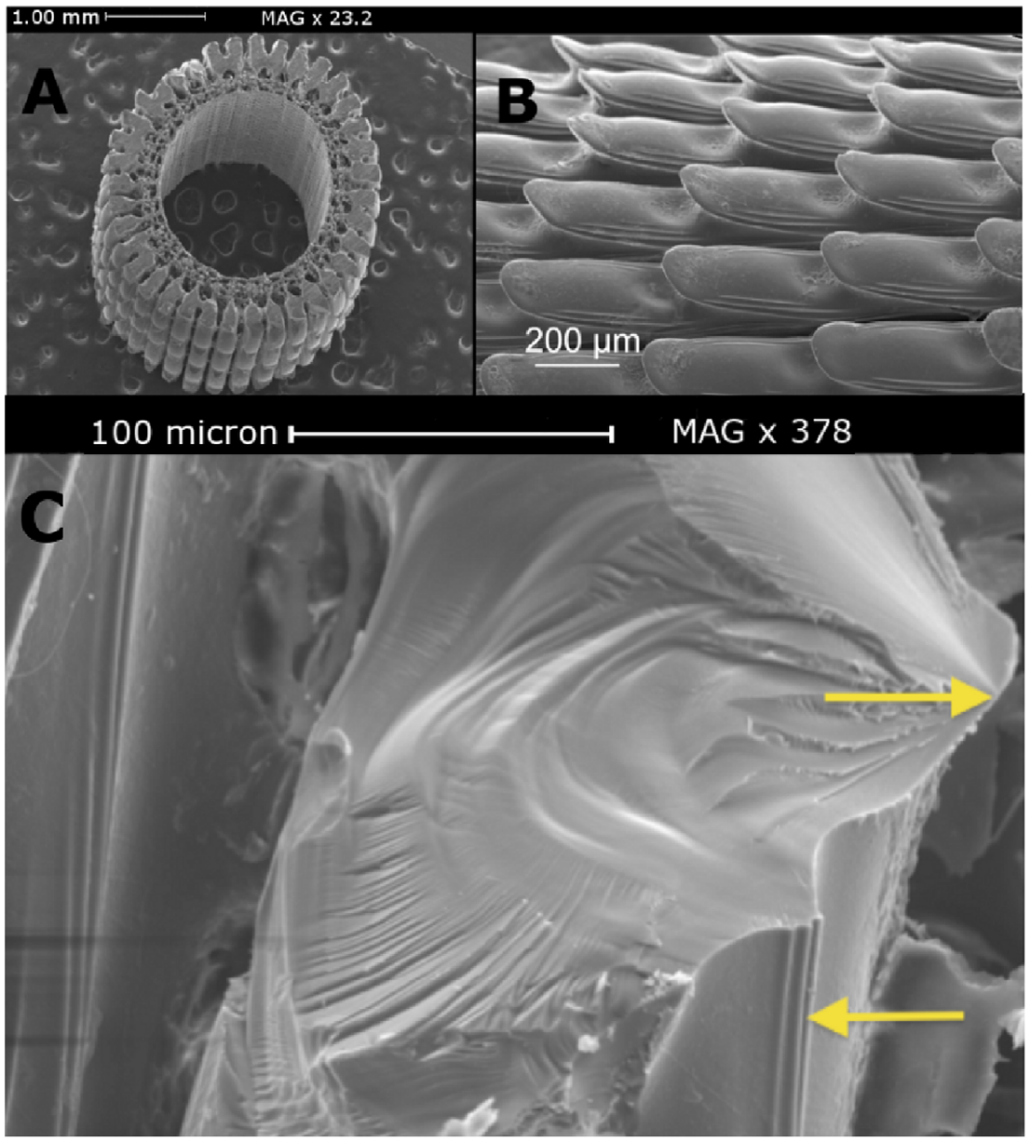
SEM micrographs of a Sea Urchin spine. A: Cross-section of the spine showing its hollow center and porous wall architecture (scale bar = 1.0 mm). B: Outer surface of the spine. Barbs point toward the spine’s tip, shown here on left (scale bar = 200 micron). C: Fracture surface of a wedge of the spine (scale bar = 100 micron). The appearance is reminiscent of fracture morphology of glass. Top arrow points to the root of crack initiation. Bottom arrow points to a feature on the external surface of the wedge, also seen in B, which identifies the external surface, and confirms that crack initiation started on the outer surface.

**Figure 2:**
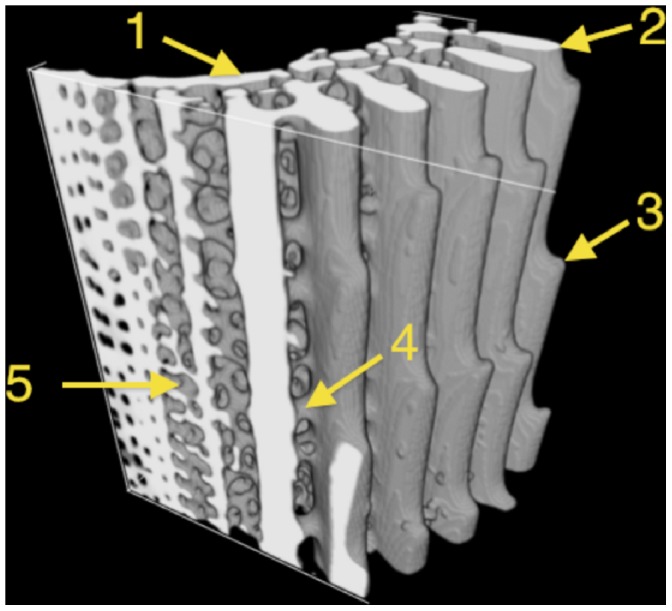
MicroCT reconstruction of a portion of the spine showing details of its internal anatomy. 1 - inner wall, 2 - wedge, 3 - barb, 4 - bridge, 5 - porous zone.

In *Centrostephanus rodgersii*, spines from near the top or sides of the round test are longest and reach up to 10 cm in length, with diameter varying from approximately 4 mm at the base reducing to 1 mm at the tip. Figure 1A shows the microstructure characteristic of all spines:- each has a wide and hollow core which is surrounded by a porous zone, extending to a set of radial wedges that form the majority of the solid cross-section. Spines also have a distinct pattern of microscopic barbs (Figure 1B) pointing toward the tip. Further details include bridges that link adjacent wedges as indicated in Figure 2. These bridges follow an irregular helical pattern around the longitudinal axis of the spine [7], [8]. The central core is comprised of a thin calcite wall incorporating a regular array of holes. Overall, the spine is highly porous with an intricate structural hierarchy.

Figure 1C shows a scanning electron microscope (SEM) image of a fracture surface through a wedge portion of a spine which was broken by bending. The morphology of the fracture surface is similar to that observed for glassy materials [9], [10] rather than a cleavage-like fracture of a single crystal. The spines are strengthened due to substitution of magnesium (Mg) for calcium (Ca) in the carbonate crystal [5]. The Mg content impedes the perfect cleavage of the calcite lattice [5] in a crack-deviating mechanism, altering the fracture behavior of the calcite.

Preliminary chemical analyses were made on polished sections of spines to search for chemical variation in the materials being investigated (see Materials and Methods). This has been done as carbonate from Sea Urchin skeletons is known to vary in chemical composition [5], [6], partly a difference between species possibly related to temperatures of growth, partly from differences between different skeletal parts in single animals, and partly from variations across single skeletal parts (for example, Mg decrease from base to tip of individual spines).

Hollow cylindrical shells are a common structure in nature, for example trabecular bones, spines, quills and plant stems. This morphology, comprised of a solid outer shell with a porous core, is advantageous and effective for mechanical efficiency and high strength-to-weight ratio. Biological cylindrical structures often fail in elastic buckling due to combined axial compression and bending loads [11]. However Urchin spines, with their single-crystal material, exhibit elastic properties as well as brittle fracture.

The abaxially and radially oriented bridges spiral around the spine’s axis, and together with the wedges act to concentrate mass to the outside radius of the spine [7]; it is therefore expected that in a direct collision on the spine axis, the force of impact would be transferred to the wedges, leaving the central cylinder unharmed. Spaces between wedges also serve to stop fractures from propagating through the structure, increasing the fracture strength of the spine beyond that of a monolithic calcite tube as the cracks must propagate separately in each wedge instead of propagating from one nucleation site to the entire cross-section [12].

To better understand the complexity of the spine’s microstructure we created a model of a Sea Urchin spine which incorporates 3D geometry based on microCT imaging (Figure 2), and anisotropic material properties (see Materials and Methods for details). Finite element analysis was used to study a model of an urchin spine through simulated mechanical deformation. While Sea Urchin spines have been studied by microCT [8], and models using simplified geometries have been constructed to study their mechanics [12], to our knowledge this is the first study to model accurate spine microstructure based on microCT imaging. By incorporating accurate, tomography-based geometry at the micron scale into the model, the detailed contribution of each hierarchical sub-structure of the spine to the overall load-bearing capacity can be examined.

We subjected the model to compression, tension and torsion loads which the spine may encounter in nature, and studied the resulting stress distributions. The stress and strain distributions that occur throughout the spine demonstrate how applied mechanical loads lead to different stress concentrations in the spine, resulting in either an elastically resilient structure, or one that snaps in brittle failure, depending on the type of load that is applied to it.

## Results and Discussion

### 1. Compression and Tension

**Figure 3:**
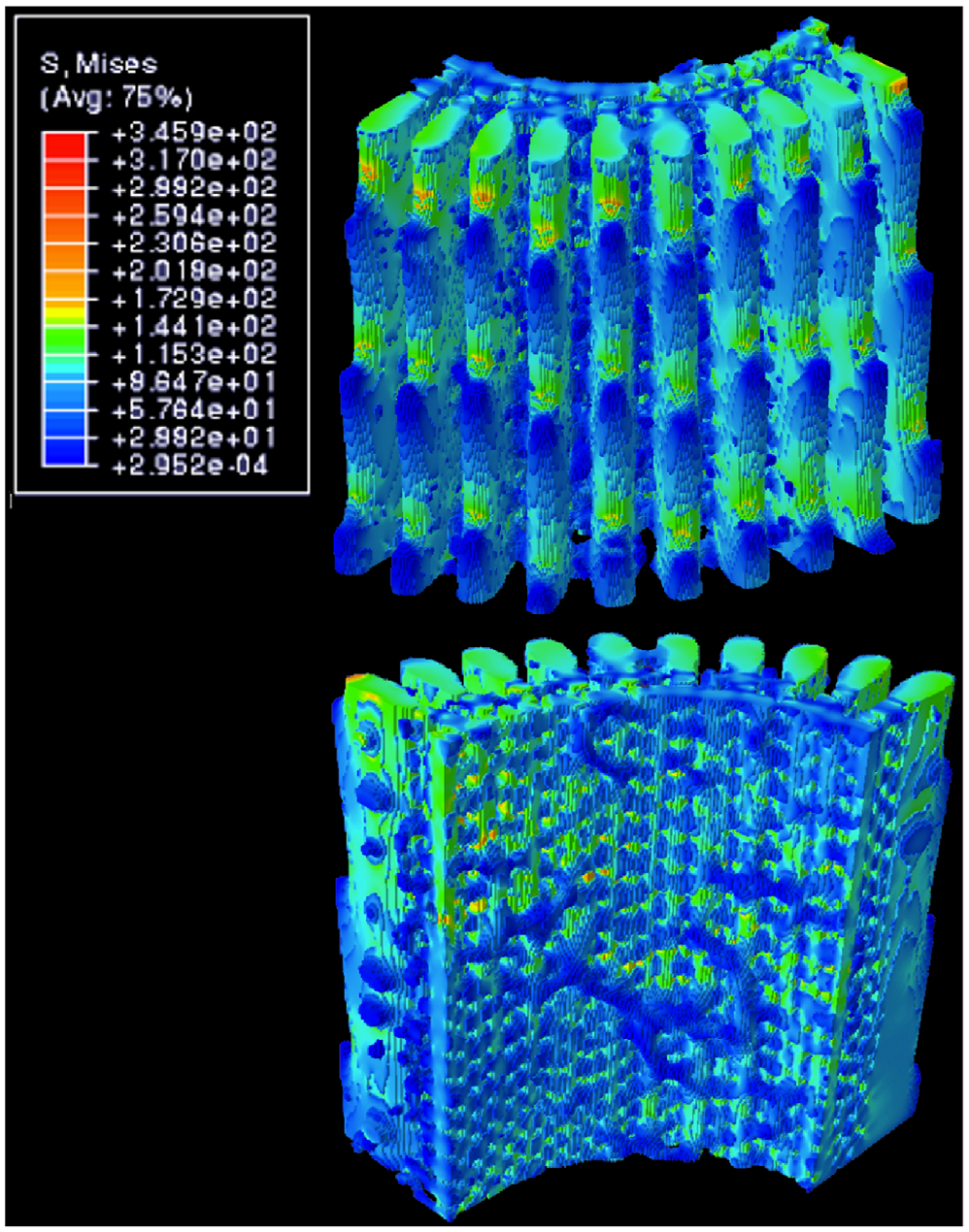
Distribution of von Mises stress under 1% applied compressive strain. The value of stress (MPa) is indicated in the insert; blue - low level, red - high level of stress. Top: outer surface of spine. Bottom: inner surface.

Compression and tension simulations gave analogous results, as expected for loads within linear elastic limits, applied along the symmetrical c-axis of the spine. Results for compression, color coded according to the level of stress, are shown in Figure 3. It can be seen that the wedges carry most of the load, and the stress in each wedge in the x-y plane appears to be constant and homogeneous over the cross-sectional surface. Barbs, being protrusions from the outer surface of the wedges, are under relatively low stress. In the inner porous zone of the spine the level of stress is most heterogeneous, evidently reflecting the complexity of the microstructure.

**Figure 4:**
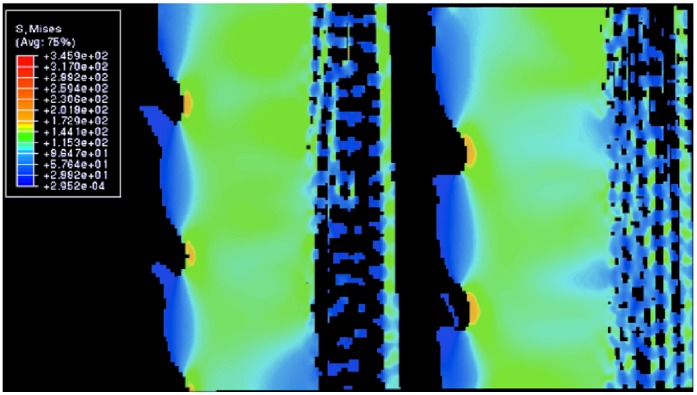
Vertical cuts through selected wedges showing internal stresses. The stress distributions here are cross-sectional cuts through some of the wedges shown in Figure 3, showing inhomogeneous stress, from low in the barbs (dark blue), to high in between the barbs (orange). Width of each image is approximately 600 micron.

Figure 4 shows vertical cuts through the centre of two wedges located midway in the model, far from spurious artifacts at the edges. In these images the barb profile is clearly visible, as well as the porous portion of the spine surrounding the hollow core. High stress concentration occurs in the small region of the wedge between the barbs, counterbalanced by lower stress zones extending axially into the body of the wedge. Figure 4 also shows four distinctive stress regions through the wedges: (i) a low stress region on the tip of the barb, (ii) a medium low stress region that extends further into the body of the barb and in the porous zone, (iii) a medium high stress region in the body of the wedge between barbs, and (iv) the high stress concentration already mentioned near the outer surface between barbs.

### 2. Torsion

**Figure 5:**
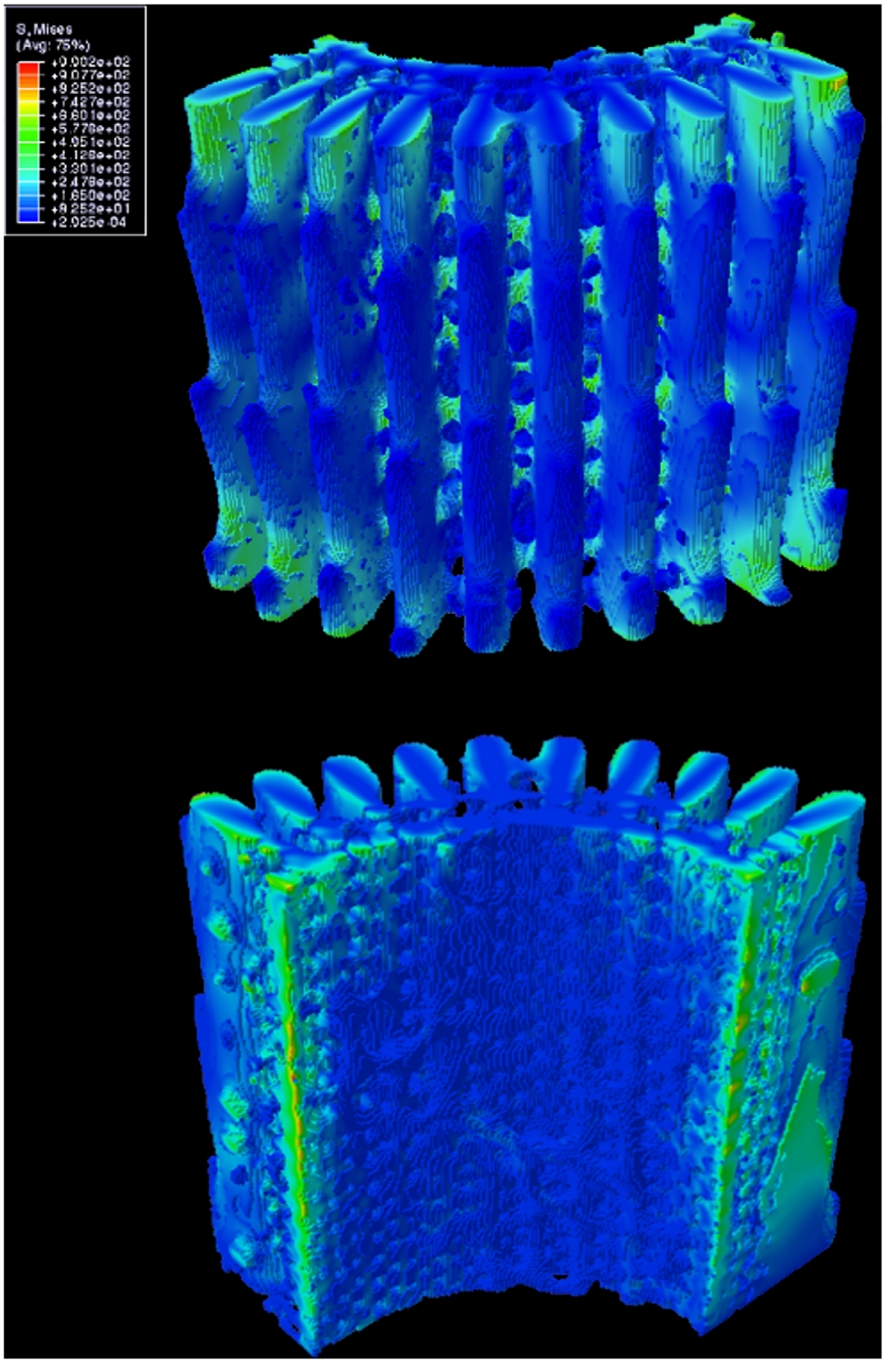
Distribution of von Mises stress under torsional loading. The value of stress (MPa) is indicated in the insert; blue - low level, red - high level of stress. Top: outer surface of spine. Bottom: inner surface.

Results of torsion loading, shown in Figure 5, provide the significant observation that there is a definite stress elevation on the bridges due to the shearing motion between the wedges as stresses applied in the xy plane cause conjugate shear stress in the yz plane. The wedges suffer relative displacement in shear along their lengths, with stress concentrating on the bridges due to their smaller cross-sectional areas. No stress is seen on the body of barbs and central cylinder. When an elastic cylinder is subjected to torsion around its longitudinal axis, the magnitude of the tangential displacement of cylinder elements in any xy cross-section is proportional to radial distance from the centre. This causes the higher stresses seen on the sides of the wedges in Figure 5, but not in the middle. In addition to the above effect, for a cylinder with trigonal symmetry, a shear strain applied in the xy plane ( = ε_23_ = ε_32_), will cause normal strain in the x-axis (due to c_24_≠0) and y-axis directions (due to c_14_≠0), but no strain in z-axis direction (due to c_34_ = 0). Note that the high stresses seen on the symmetry planes in Figure 5 are due to edge artifacts as boundary conditions there prevent displacements in the y and x axes.

### 3. Implications of the Model

The body of the spine is not a solid cylinder, but an assembly of wedges fanning out from the centre, interconnected by bridges. Thus, under torsion loading, the bridge appears to be the only substructure that resists the relative shearing motion of the wedges. Without these bridges the spine structure would exhibit high compliance to shearing, leading to premature structural failure. This result further supports the claim made by Stock et al [7] who discussed the significance of bridges between wedges, with wedges serving the purpose of concentrating mass to the outside radius of the spine.

**Figure 6:**
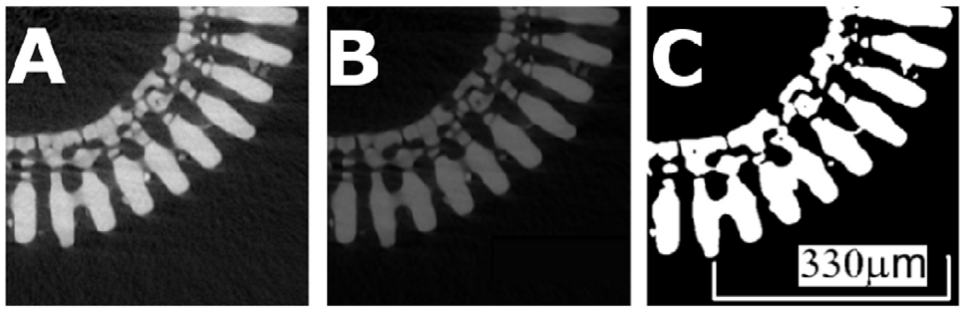
Contrast improvement of microCT image. A: original microCT image, B: noise reduction, and C: anisotropic diffusion filtering.

The majority of high stress-concentration points in Figures 3 and 4 (modelled compressions) are situated in the wedges, especially near the barbs. These would most likely be locations where structural failure will initiate due to formation of cracks that propagate from the highly stressed surface points. Thus, the explanation of Burkhardt et al [12] in regards to the gap between wedges serving the purpose of limiting propagation of cracks seems well-founded. We have found that the wedges and the central cylinder take part in bearing stress under compressive loads. However, it is clear that the wedges act as the main support for the spine and distribute the majority of load along its body. Although bridges and barbs are attached to the wedges, they have virtually no load bearing capacity or function.

**Table 1:** Elastic constants for single crystal of calcite [21] used in the finite element model.


Component	C_11_	C_33_	C_44_	C_12_	C_13_	C_14_
Elastic Stiffness (GPa)	149.4	85.2	34.1	57.9	53.5	−20

### 4. Limitations of the Model

Our simulation results were obtained for a solid of uniform composition that was chosen as a simple, first-approximation model for mechanical analysis. However minor radial composition gradients exist (see Materials and Methods) that have two direct effects. First, there will be corresponding minor gradients in the magnitudes of elastic constants, leading to small stress variations. Second, if the substitution of magnesium for calcium introduces compressive hydrostatic stress component into the outer layers of the wedge, this could lead to increased resistance to fracture by neutralizing the effect of surface micro-cracks, analogous to the classical case of fracture toughening of glass by replacing sodium with potassium ions [19].

**Figure 7:**
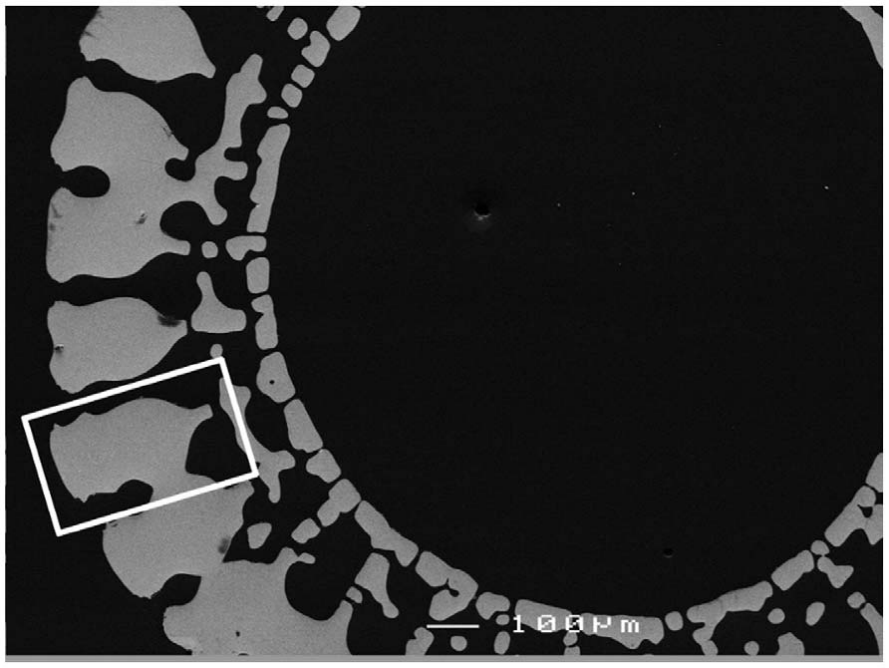
SEM micrograph of spine transverse section. White rectangle indicates single wedge area used for chemical investigation.

**Figure 8:**
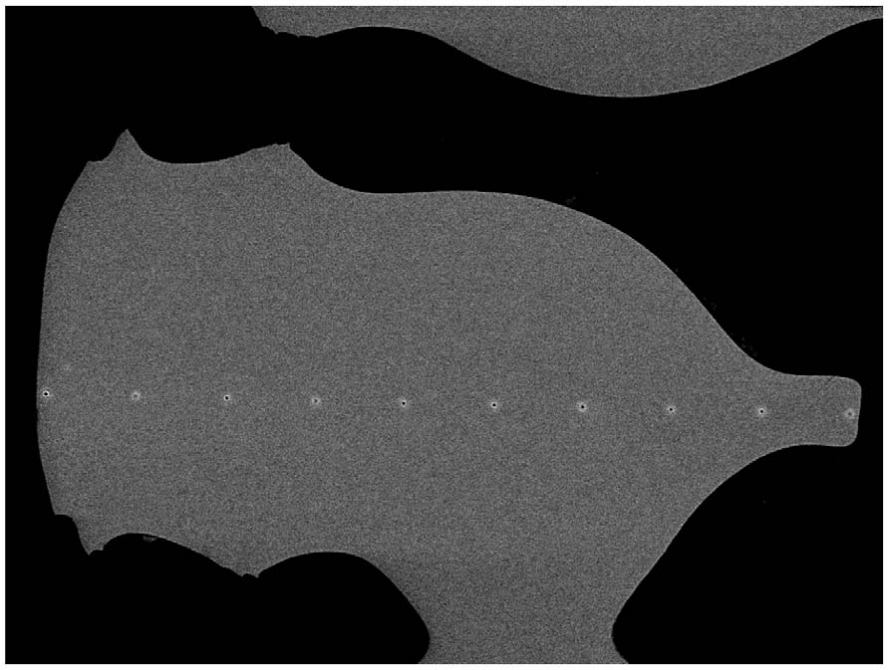
SEM micrograph of the area after chemical analysis. Note the line of 10 analysis spots at a spacing of 38 micrometers along a line from the tip to the base of this wedge.

**Figure 9:**
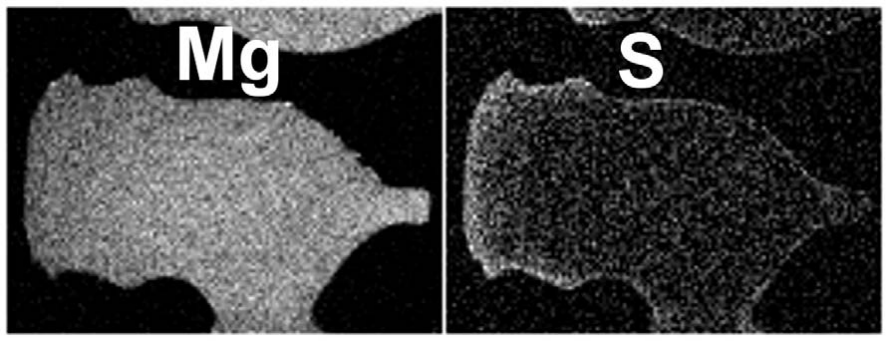
Maps of X-ray intensities. Intensities of Mg (left) and S (right) detected across the area used for chemical analyses. In both maps, brightest regions are those with highest abundance.

Furthermore, we have not taken into account what effect air-drying may have on the mechanical properties of the spine. If existing, the embedded small amounts of glycoprotein should be modeled separately as soft phase inclusions in a composite material comprising a hard single-crystal matrix. This is a worthy topic of research and calls for a separate scientific study.

**Figure 10:**
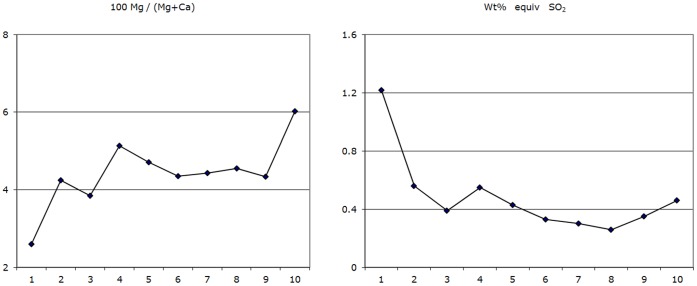
Chemical analyses. Representative results of analyses made along the line indicated in Figure 8. Some variation in composition was measured from the wedge tip (point 1 plotted at left) to the base (point 10 plotted at right), reinforcing the trends from X-ray-intensity maps of Figure 9.

While this study has focused on quasi-static mechanical loads, it could be revealing to look at the effect of dynamic impact loads on spines. Further avenues of research could look at spines from several species of urchin to gauge the contribution of different morphologies to overall strength and mechanical behavior. Locomotion and sensing functions require the spines to withstand mild compressive forces with some elasticity. Indeed, the brittleness of the single-crystal calcite is tempered by the inclusion of minute amounts of organic material. When the urchin is impacted by a foreign object, the spines protect the test by absorbing the impact energy and snapping in brittle failure [1]. This requires the spines to fracture in tension under bending loads. When the spines pierce an attacking predator, they must have high longitudinal compressive strength to withstand the initial impact, but subsequently snap in brittle failure due to bending or torsion loads while remaining embedded in the predator.

### Conclusions

We have characterised in detail the nature of spines in one species of Sea Urchin. This primarily involved imaging and microCT analysis of spine morphology, but also included preliminary analysis of some aspects of the chemical composition. MicroCT data was subjected to finite element analyses to investigate a range of applied load conditions, then to search for patterns of stress concentrations. We have discussed both implications and limitations of our investigations.

Bejan’s constructal theory [13], [14] states that the optimal distribution of imperfections is a principle that underlines efficiency of form in nature, and that given time and the ability to change, systems organize themselves in a way which maximizes efficiency of flow. In the case of the Sea Urchin, the spine’s structure, with its intricate barbs, wedges and bridges that act as mechanical support, contributes to its strength in bending [7], [8]. In this case flow is not of fluid or heat, but rather of stress [15], and it seems that the urchin spine’s microstructure may have evolved such that certain stress concentrations occur in response to various mechanical loadings. It is important to note that such evolutionary adaptations do not imply that the morphology is in any way ideal. However, the spine’s high porosity, and the way in which its variation distributes stresses throughout its structure in response to applied loads, result in a structure that is strong and lightweight, especially considering the brittleness of the constituent material [16]–[18].

## Materials and Methods

### 1. Sample Origin

This study focuses on the mechanical properties of the solid parts of the spines of *Centrostephanus rodgersii*, extracted from the Sea Urchin collected live in Batemans Bay, NSW, Australia. No specific permits were required for the described field studies. The beach where the samples were collected is public, and this species of Sea Urchin is not endangered or protected. The skeletal structure was air dried for more than two years, causing the organic tissues and membranes to naturally decay during storage in dry ambient air.

### 2. MicroCT Imaging and Finite Element Analysis

Segments of Sea Urchin spine, approximately 20 mm long, were scanned using microCT at a voxel resolution of 2 micron (focused electron beam, polychromatic X-ray beam via bremsstrahlung of 80kV/0.1 mA, pre-filtered with a 1 mm CaCO_3_ wafer to minimize the phenomenon of beam hardening). The samples were rotated through 360° in angular increments of 0.2°, producing 1,800 slices of 20,482 pixels. The reconstructed three dimensional tomogram was binarized and processed using an anisotropic diffusion filter to enhance edge detection using Mango (Medial Axis and Network Generation, Australian National University and the Friedrich-Alexander-Universität Erlangen-Nürnberg), as seen in Figure 6.

The 3D microCT image was converted into a finite element mesh by the direct voxel conversion method; a discussion of the method is detailed elsewhere [20]. The commercial FEA suite ABAQUS (Dassault Systemes, France) was used for model pre-processing, simulation and results post-processing. Voxels were binned by a skip rate of 1, resulting in a voxel resolution of 4 micron; reduction in resolution was necessary due to computational limits. Each voxel was converted into an 8-node hexahedral element. The spine was assumed to be an axially symmetric, cylindrical body, so one quarter of the imaged spine was modeled to keep within computational limits. Thus the highly complex, porous microstructure of the spine was modeled in the finite element mesh.

As has been shown by many microscopic, analytical and X-ray diffraction investigations (e.g. [5]) the mineral matter in the spines is Mg-bearing rhombohedral calcite which forms one single crystal continuous throughout the complex porous solid. An invariant anisotropic stiffness tensor was therefore assigned to every solid element in the model, corresponding to the known constants for single-crystal calcite with crystallographic c-axis parallel to the Urchin spine [21].

We have verified the single-crystal nature of the spines by optical polarized microscopy and electron diffraction in TEM in our laboratory. The crystal system is trigonal, with symmetry elements: 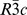 [22]. Since collagen (type I) also possesses trigonal symmetry [23], it would be interesting to speculate whether the trigonal crystal structures are coincidental or not. The biochemistry of these proteins is well characterized and their associated inorganic minerals are commonplace in the materials world. Politi et al. studied the transformation of amorphous calcium carbonate into calcite [24] and also reported on the mechanism of crystal formation during spine regeneration [25].

Anisotropic single-crystal properties were assigned to model elasticity in the unique structure of the crystalline spine material. The spine, with crystal [c] axis parallel to its long axis, is described by the matrix of elastic constants shown in Table 1 [21] in shorthand notation. The stress-tensor components, with generalized Hooke’s law, were used to calculate the principal stresses.

Symmetry boundary conditions were set on the vertical surfaces of the model to simulate cylindrical symmetry. Three load cases were modeled by applying the following quasi-static loads to the top surface nodes: (i) 1% displacement in the z-direction (along the spine axis) in compression, (ii) similarly in tension, and (iii) torsion modeled as a 1° twist applied to the top surface around the centre axis of the cylinder.

Boundary conditions for the quarter segment were set to:

- for cases (i), (ii) and (iii) bottom surface: no rotations, no displacement in z-axis, unrestricted displacements in x and y-axis (to allow for Poisson’s effect)
- for cases (i) and (ii) top surface: no rotations, unrestricted displacements in x- and y-axis (to allow for Poisson’s effect), ε_33_ = −0.01 strain for compression, or ε_33_ = +0.01 strain for tension
- for cases (i) and (ii) vertical x−z surface: no rotations, no displacement in y-axis, unrestricted displacements in x and z-axis
- for cases (i) and (ii) vertical y−z surface: no rotations, no displacement in x-axis, unrestricted displacements in y and z-axis
- for case (iii) top surface: no rotations around x and y-axes, no displacement in z-axis direction, 1° rotation around z-axis
- for case (iii) x−z surface: unrestricted displacements in x and y-axes
- for case (iii) y−z surface: unrestricted displacements in x and y-axes

### 3. Chemical Analysis

Analyses were made using an Energy-Dispersive X-ray Spectrometer (EDS) fitted to the JEOL SEM used to image the spines. The raw data could be roughly assessed by inspecting the EDS spectra, but for better reliability over 50 chemical analyses were made from micron-sized regions, each analysis being processed from spectra via the procedures known in general as ZAF correction [26] using verified standards. In addition, some uncorrected chemical maps were made to reveal spatial distribution of X-ray intensities, a technique widely used to indicate chemical variation.

In longitudinal sections, no chemical variability from spine base to tip could be discerned. However, variability was recorded in a transverse section. Figure 7 shows a micrograph of this specimen recorded using SEM. The area used for X-ray mapping is indicated by the rectangle marked and detailed in Figure 8. X-ray maps with higher intensity indicating increased chemical abundance of the corresponding element, are shown for Mg and S in Figure 9. The variation extends across the spine wedge. Other elements measured do show variations not inside the wedge but restricted to the hard-spine surfaces and dismissed as related to surface coatings or contaminations.

To further characterize chemical variation, corrected analyses were made along a line from the tip to the base of the same wedge (see ten analysis marks in Figure 8). In general the analyses confirm the trends revealed from mapping, for example while the composition of the carbonate is mainly 4%–5% molar MgCO_3_, it appears to vary from approx 2.55% to 6% along the line analyzed. Plots of Mg, S, Ca and Mg/(Mg+Ca) are shown in Figure 10. There is clearly a decrease in Mg towards the wedge tip. There is also a clear increase in S at the wedge surface, but both maps and profiles suggest this is spatially restricted compared to the Mg variation.

These chemical analyses indicate variation in composition of the carbonate with some trends identified for one area in detail. However, much more careful analytical work is required to definitely establish the patterns and examine whether they apply to all wedges and spines. Further analyses along the spine length are also warranted.
